# Precocious puberty in an infant with hepatoblastoma: a case report

**DOI:** 10.1186/1752-1947-5-422

**Published:** 2011-08-30

**Authors:** Usama Al-Jumaily, Ibrahim Sammour, Fadi Al-Muhaisen, Fatenah Ajlouni, Iyad Sultan

**Affiliations:** 1 Department of Pediatric Oncology, King Hussein Cancer Center, Queen Rania Al Abdullah St, Amman, 11941, Jordan; 2 Department of Radiology, King Hussein Cancer Center, Queen Rania Al Abdullah St, Amman, 11941, Jordan

## Abstract

**Introduction:**

The syndrome of isosexual precocious puberty associated with primary malignant hepatic tumors is rare. All previously reported cases in the literature are old and prognosis was grim.

**Case presentation:**

We present the case of a 15-month-old Asian male baby who presented with precocious puberty associated with hepatoblastoma. Serum concentrations of alpha-fetoprotein and free testosterone were elevated, as was beta human chorionic gonadotropin hormone. He was treated with six courses of chemotherapy and underwent surgery. His surface markers as well as free testosterone level returned to normal during therapy. The child has now been off therapy for 18 months with no evidence of tumor recurrence at follow-up.

**Conclusion:**

Virilizing hepatoblastoma is rare and reported with poor outcome, but the development of new chemotherapeutic agents and complete surgical resection are promising.

## Introduction

Hepatoblastoma is the most common pediatric hepatic tumor. Using the current modalities of treatment, non-metastatic hepatoblastoma usually carries a favorable prognosis if completely resected. The tumor typically secretes α-fetoprotein (AFP) which is a useful marker for management and follow-up. On rare occasions, hepatoblastoma is associated with beta human chorionic gonadotropin hormone (β-hCG) secretion [1].

Paraneoplastic features of hepatoblastoma are not uncommon at presentation and include thrombocytosis and increased alkaline phosphatase [2]. Occasionally, isosexual precocious puberty was reported in boys with hepatoblastoma [1,3-15]. While most cases were reported in the 1980s, we believe documenting the response of similar cases to treatment in the modern era is important.

### Case presentation

A 15-month-old Asian male baby presented to our center with precocious puberty and a hepatic mass. He was a product of cesarean section at 37 weeks of gestation. He had meconium aspiration at birth but had no history of hypoglycemia. His birth weight was 3.75 kg (in the 75^th ^centile). At the age of eight months his mother noticed enlarging genitalia with sparse pubic hair and changes in his voice. These symptoms progressed over time and six months later, he started to have persistent fever and abdominal distension. A right upper quadrant mass was palpable at that time, so he was referred for evaluation. His family history was significant for an older sibling who died after being diagnosed with Wilms' tumor at the age of ten years and for a grandparent who died of lung cancer. There was no family history of overgrowth syndrome or other pediatric tumors. His parents were not related by blood.

**Figure 1:**
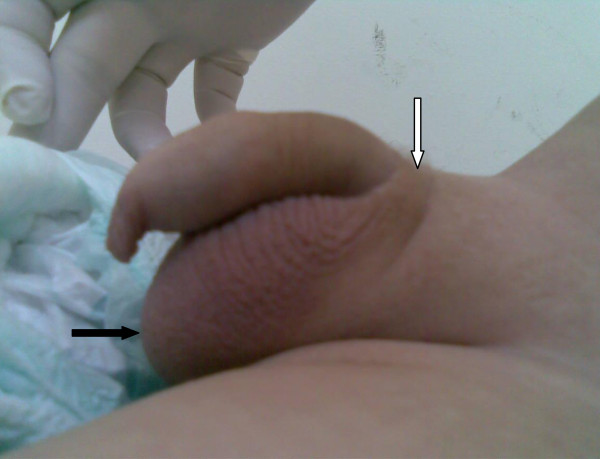
**Photograph of patient's genitalia at 15 months of age**. Shows large pigmented scrotum (light arrow), scant pubic hair [not clear on the picture] (dark arrow) and penile enlargement (10 cm stretched length).

**Figure 2:**
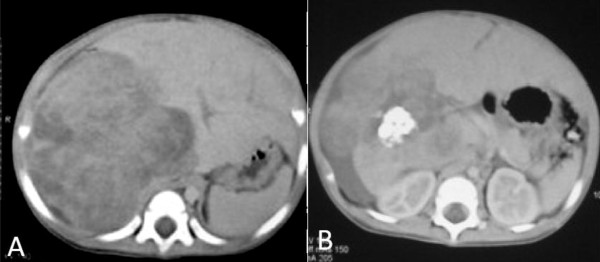
**CT scan of the liver showing the hepatic tumor**. (A) Involvement of the right hepatic lobe; (B) area calcification and IVC extension.

At the time of presentation, his height was 82 cm (in the 90^th ^centile for age) and his weight was 12.45 kg (in the 95^th ^centile). There was no evidence of hemihypertrophy or dysmorphic features. He had a large-for-age, uncircumcised penis that measured 10 cm from the base with a large thick scrotum and scarce suprapubic hair (Tanner stage was III, as seen in Figure 1). A workup revealed a bone age of two years and eight months and blood investigations showed elevated AFP (356,474 ng/mL, normal for age < 12 ng/mL), β-hCG (13.7 mIU/mL, normal for age < 2 mIU/mL), and free testosterone (10 pmol/L, normal for age 0.09-5.4 pmol/L) along with thrombocytosis (platelet count of 1244 × 10^3^/μl). An abdominal ultrasound revealed a right hepatic lobe mass. A computed tomography (CT) scan (Figure 2) showed a large heterogeneously enhancing right hepatic lobe measuring 10.5 × 11.5 cm and involving mainly segment VII and parts of segments V, VI and VIII in keeping with a 2-sector involvement with inferior vena cava (IVC) extension almost reaching his right atrium (PRETEXT II-V3). A metastatic work-up showed no metastasis to his lung. Other hormonal laboratory tests, including serum prolactin (16.69 ng/mL, normal for age 4.1-18.4 ng/mL), androstenedione (0.6 ng/mL, normal for age 0.3-3.5 ng/mL), dehydroepiandrosterone sulfate (0.182 μmol/L, normal for age 0.9-11.7 μmol/L), thyroid stimulating hormone (3.7 mIU/L, normal for age 0.3-5 mIU/L), free thyroxin (1.4 ng/dL, normal for age 0.71-1.85 ng/dL), free triiodothryronine (0.44 pg/mL, normal for age 1.45-3.48 pg/mL), follicular stimulating hormone (0.5 IU/L, normal for age 1-8 IU/L), luteinizing hormone (1.08 mIU/mL, normal for age 5-12 mIU/mL), and a gonadotrophin releasing hormone test, were all within normal range for age. Brain magnetic resonance imaging did not detect any abnormality in his brain or pituitary gland. A testicular ultrasound (US) appeared normal and unremarkable. A testicular biopsy was not done.

**Figure 3:**
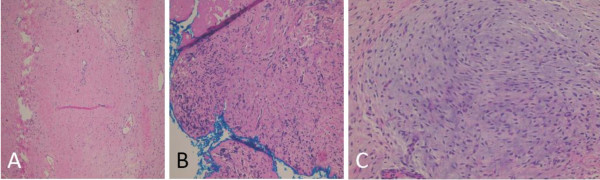
**Postsurgical histopathology showing chemotherapy effect**. (A) Fibrosis, hyalinization and myxomatous changes seen in 30% of the tumor size; (B) free surgical resection margin; (C) background of mixed epithelial mesenchymal hepatoblastoma with a predominant epithelial subtype.

Our patient was treated with four cycles of preoperative chemotherapy (cisplatin, alternating with carboplatin/doxorubicin). The tumor showed a good partial response with more than 50% reduction in the size of the mass and a resolved IVC thrombus. Doppler US confirmed patency of the hepatic, portal and IVC veins. During therapy, we observed a decline in the level of free testosterone (Table 1) with an arrest of further virilization. Our patient underwent surgery with complete excision of the tumor, followed by two additional chemotherapy cycles containing cisplatin. A pathology review (Figure 3) showed residual hepatoblastoma with 90% necrosis. The viable part showed fetal histology and stained positively for Hep Par1 and CD34. The resection margins were free of tumor and there was absence of blood vessel invasion. Laboratory and radiologic evaluation revealed no evidence of tumor recurrence one and a half years after completion of therapy. Our patient was 33 months old on the last visit and no further development of secondary sexual signs were elicited. His height was 96 cm (between the 50^th ^and 75^th ^centile) and his weight was 14.2 kg (at 50^th ^centile).

**Table 1:** Results of laboratory investigations during and after treatment

	AFP (ng/mL)	β-hCG (mIU/mL)	Free testosterone (pmol/L)
**Normal values for age**	< 12	< 2	0.09-5.4
**At time of presentation**	356,474	13.7	10
**After first chemotherapy cycle**	78,077		
**After second chemotherapy cycle**	7062	< 2	
**After fourth chemotherapy cycle**	67.52		4.5
**After surgery**	10.6	< 2	
**At end of therapy**	5.1	< 2	0.1
**Follow-up six months after end of therapy**	3.76	< 2	

## Discussion

Isosexual precocious puberty due to a virilizing hepatoblastoma is a rare but well documented occurrence [1,3-15]. Cases have been confined to boys, generally below three years of age, who have usually presented with accelerated skeletal growth and virilization [4]. Hepatic enlargement at presentation has been invariable.

Two hypotheses explaining androgen secretion are suggested in the literature: ectopic testosterone secretion and secondary testosterone secretion. The first theory suggests excess secretion of testosterone by neoplastic cells [4], while the second theory suggests secondary stimulation of the testes by β-hCG [3,4,7,8]. While it remains difficult to speculate as to which may be the correct theory, it is interesting to notice that virilization occurred in all reported children with hepatoblastoma in association with elevated β-hCG levels, supporting the role of this hormone in excessive androgen production. Central and secondary precocious puberty such as hypothyroidism, premature adrenarche and congenital adrenal hyperplasia were first excluded by performing the appropriate tests. Together with elevated β-hCG levels and the findings of imaging studies, the correct diagnosis was suspected and proper therapy was initiated.

The majority of the previous cases were reported more than two decades ago, making it difficult to judge the outcome of virilizing hepatoblastoma, taking into consideration the inferior quality of imaging techniques, surgical techniques and chemotherapeutic regimens. As a matter of fact, it was suggested that virilizing hepatoblastoma carried a worse outcome when compared to nonvirilizing tumors, alluding to a unique biological setup [8]. Our patient's response to therapy was favorable. His initial presentation with IVC thrombosis had put him at a higher risk for local failure; however, the favorable response to chemotherapy and complete resection is reassuring.

## Conclusions

Although isosexual precocious puberty with hepatoblastoma is rare and carries a poor outcome (as reported in most cases), results due to developments in chemotherapeutic agents and complete surgical resection are promising.

## Consent

Written informed consent was obtained from the patient's legal guardian (father) for publication of this case report and any accompanying images. A copy of the written consent is available for review by the Editor-in-Chief of this journal.

## Competing interests

The authors declare that they have no competing interests.

## Authors' contributions

UA interpreted the patient data and was a major contributor to the writing of the manuscript. IS and FAM collected the clinical data. FA obtained and interpreted radiological studies. UA and IS reviewed the literature. IS gave approval for the final manuscript. All authors read and approved the final manuscript.
